# LINE-1 hypermethylation in white blood cell DNA is associated with high-grade cervical intraepithelial neoplasia

**DOI:** 10.1186/s12885-017-3582-0

**Published:** 2017-08-30

**Authors:** Martina Barchitta, Annalisa Quattrocchi, Andrea Maugeri, Carolina Canto, Nadia La Rosa, Maria Antonietta Cantarella, Giuseppa Spampinato, Aurora Scalisi, Antonella Agodi

**Affiliations:** 1 Department of Medical and Surgical Sciences and Advanced Technologies “GF Ingrassia”, University of Catania, via S. Sofia, 87, 95121 Catania, Italy; 2 Oncopath s.r.l, Floridia, SR Italy; 3 Unità Operativa di Screening Ginecologico, Azienda Sanitaria Provinciale 3, Catania, Italy

**Keywords:** LINE-1 methylation, Global DNA methylation, Hypermethylation, Cervical cancer, Cervical Intraepitelial Neoplasia, ROC curve analysis, Pyrosequencing-based methylation analysis, Prevention

## Abstract

**Background:**

Long Interspersed Nuclear Elements-1 (LINEs-1) methylation from white blood cells (WBCs) DNA has been proposed as biomarker associated with different types of cancer. The aim of the present study was to investigate the degree of WBCs LINE-1 methylation, according to high-risk Human Papilloma Virus (hrHPV) status in a healthy population, and the association with high-grade Cervical Intraepithelial Neoplasia (CIN2+) in hrHPV positive women.

**Methods:**

Women with abnormal cervical cells were enrolled and classified by histological diagnosis and hrHPV infection. A structured questionnaire was used to obtain information on socio-demographic variables and lifestyle factors. LINE-1 methylation level in WBCs was measured by pyrosequencing-based methylation analysis after bisulfite conversion.

**Results:**

Among 252 women diagnosed with normal cervical epithelium, with regard to LINE-1 methylation level no significant difference was observed between hrHPV positive and hrHPV negative women, also adjusting for known risk factors of infection. The association between WBCs LINE-1 methylation and CIN2+ status was analyzed in hrHPV positive women. The median value of LINE-1 methylation levels was higher in cases (CIN2+) than in controls (75.00% versus 73.17%; *p* = 0.002). For a one-unit increase in LINE-1 methylation level, the odds of being diagnosed with CIN2+ increased by 10%, adjusting for known factors related to LINE-1 methylation (adjOR: 1.10; 95% CI:1.01–1.20; *p* = 0.032). The Receiver-Operating Characteristic (ROC) curve analysis identified the cut-off value of 73.8% as the best threshold to separate cases from controls (sensitivity: 63.4% and specificity: 61.8%).

**Conclusions:**

LINE-1 methylation status in WBCs DNA may represent a cost-effective and tissue-accessible biomarker for high-grade CIN in hrHPV positive women. However, LINE-1 hypermethylation cannot be considered specific for cervical cancer (CC) and a model based solely on LINE-1 methylation levels has limited performance. Further investigations are necessary to propose and validate a novel methylation biomarker panel, based on LINE-1 methylation and other differentially methylated regions, for the screening of women at risk of CC.

## Background

Cervical cancer (CC) is the fourth most common cancer and an important cause of death worldwide [1]. CC arises through a multistage process of carcinogenesis, and persistence of high risk Human Papilloma Virus (hrHPV) infection represents the major etiological factor for neoplasia development [2–4], through the progression of precursor lesions (i.e. Cervical Intraepithelial Neoplasia, CIN) to invasive cancer [5, 6]. Among the putative molecular alterations leading to morphological modifications, aberrant DNA methylation might be an important event in cervical carcinogenesis [7, 8]. DNA methylation at specific CpG sites in hrHPV or in human genes has shown the potential for the detection of CIN2+ and some biomarkers have been proposed [8–15].

Methylation in repetitive elements has been shown to correlate with global genomic DNA methylation, as a result of the high occurrence of these sequences throughout the genome [16]. Methylation of Long Interspersed Nuclear Elements - 1 (LINEs-1) has been proposed as a surrogate marker for estimating the global DNA methylation levels in cancer tissues [17] and in peripheral blood samples [18]. Furthermore, a systematic review and meta-analysis reported that LINE-1 methylation levels were significantly lower in cancer patients compared to healthy controls in tissue samples but not in blood [19]. However, several studies have shown that LINE-1 hypo- and hyper-methylation from white blood cells (WBCs) DNA are associated with different types of cancer [20–32]. Particularly, evidence from women recruited in the “Prognostic Significance of DNA & Histone Methylation” project showed that a higher degree of LINE-1 methylation in peripheral blood mononuclear cells (PBMCs) was associated with lower risk of CIN2+ [8]. Although susceptibility to hrHPV related carcinogenesis may also be an epigenetically modified process, further studies are needed to clarify the association between HPV status and LINE methylation [33].

The aims of the present study were to investigate the degree of WBCs LINE-1 methylation, by bisulfite pyrosequencing, in a population of women referring to a cervical cancer screening program and to evaluate the association with their hrHPV status, and with high-grade CIN in the hrHPV positive women subgroup.

## Methods

### Study design

During a three-years period (from 2013 to 2015), all women diagnosed with abnormal PAP test, referring to the cervical cancer screening unit (Unità Operativa di Screening Ginecologico) at the Azienda Sanitaria Provinciale (ASP 3) in Catania (Italy), for further examination by colposcopy and biopsy, were invited to participate in a cross-sectional study.

The study protocol was approved by the ethics committee of the involved Institution (CE Catania 2; Prot. N. 227/BE and 275/BE) and performed according to the Declaration of Helsinki. Participants were fully informed of the purpose and procedures of the study, and a signed written consent was obtained.

Women were classified by histological diagnosis and tested for hrHPV (hrHPV16, 18, 31, 33, 35, 39, 45, 51, 52, 56, 58, 59, and 68) using digene HC2 HPV DNA Test (Qiagen, Italy). Thus, women were classified as hrHPV positive if they were infected with any of the thirteen hrHPV types, otherwise women were classified as hrHPV negative. Notably, the specific HPV genotype is not provided by the test.

Women who tested positive for hrHPV were further classified as cases (CIN2+: CIN2, CIN3 or *carcinoma* in situ - CIS) or controls (≤CIN1: CIN1 or normal cervical epithelium), according to the histological result.

A structured questionnaire was used by trained epidemiologists to obtain information on socio-demographic variables and lifestyle factors. Women were classified into two categories of educational level: low-medium (primary school, i.e., ≤8 years of school) and high education level (high school education or greater, i.e., >8 years of school). Body mass index (BMI) was calculated based on criteria from the World Health Organization [34].

### DNA extraction and methylation analysis

Genomic DNA was extracted from whole blood using the Illustra blood genomic Prep Mini Spin Kit (GE Healthcare, Italy) according to the manufacturer’s protocol. LINE-1 methylation level in WBCs was measured by pyrosequencing-based methylation analysis, using the PyroMark Q24 instrument (Qiagen, Italy), as previously reported [35]. Briefly, bisulfite conversion and clean-up of DNA for methylation analysis of 30–40 ng of WBCs DNA were completed using the EpiTect Bisulfite Kit (Qiagen, Italy) and the converted DNA was eluted in 20 μl of Eluition Buffer.

PCR was conducted in a reaction volume of 25 μl, using the PyroMark PCR Kit (Qiagen, Italy). According to the manufacturer’s instructions, each reaction mixture contained 1.5 μl of bisulfite-converted DNA, 12.5 μl of PyroMark PCR Master Mix 2X, containing HotStartTaq DNA Polymerase, 2.5 μl of Coral Load Concentrate 10X, 2 μl of the forward primer (5′-TTTTGAGTTAGGTGTGGGATATA-3′) and the reverse-biotinylated primer (5′-biotin-AAAATCAAAAAATTCCCTTTC-3′) (0.2 μM for each) [36]. HotStart PCR cycling conditions were 1 cycle at 95 °C for 15 min, 40 cycles at 94 °C for 30 s, 50 °C for 30 s, and 72 °C for 30s, and a final extension at 72 °C for 10 min. Then, the PCR product underwent pyrosequencing using 0.3 mM of the sequencing primer (5′-AGTTAGGTGTGGGATATAGT-3′). All runs included 0% and 100% methylated human DNA as positive controls and a non­template control. Any failed LINE-1 methylation assays were excluded from the statistical analysis.

The degree of methylation was expressed for each DNA locus as percentage of methylated cytosines over the sum of methylated and unmethylated cytosines. The degree of LINE-1 methylation was reported for each locus as well as the average percentage of methylation of the three evaluated CpG sites (GenBank Accession No. X58075).

### Statistical analyses

Statistical analyses were performed using the SPSS software (version 22.0, SPSS, Chicago, IL). Descriptive statistics were used to characterize the population using frequencies, means ± standard deviations (SDs), median values and interquartile ranges (IQRs). The two-tailed Chi-squared test was used for the statistical comparison of proportions, whereas continuous variables were tested using Student’s *t* test.

The Kolmogorov-Smirnov test was performed to determine whether LINE-1 methylation levels were normally distributed. Accordingly, median LINE-1 methylation levels were compared, between case and control groups, using the Mann–Whitney U test. Correlation between LINE-1 methylation level and continuous variables was also evaluated using Pearson correlation coefficient.

In order to measure the strength of the association between categorical variables, the crude odds ratios (ORs) and the corresponding 95% confidence intervals (95% CIs) were computed. Unconditional multivariable logistic regression analyses were used to evaluate the association between the degree of LINE-1 methylation, hrHPV infection and CIN status. The analyses were adjusted for age (continuous), BMI (continuous), smoking status (current smokers vs non-smokers/former smokers), and parity (< 1 live births vs ≥ 1 live births). The adjusted ORs with the respective 95% CIs were reported. A *p* value <0.05 was considered statistically significant in all performed analyses.

The Receiver-Operating Characteristic (ROC) curve analysis was performed in order to separate cases from controls, according to mean LINE-1 methylation percentage. Area Under the Curve (AUC) and 95% CIs were calculated to assess the performance (sensitivity and specificity) of the test for each methylation value. To determine the optimal threshold of LINE-1 methylation level, suitable to distinguish cases from controls, the point on the ROC curve with the shortest distance value from the top left corner (point: 0,1) was calculated using the formula [(1 – sensitivity)^2^ + (1 – specificity)^2^] [37].

## Results

Overall, 539 women with abnormal PAP test were classified by histological diagnosis and tested for hrHPV. Among these, 252 were diagnosed with normal cervical epithelium (46.7%), 160 CIN1 (29.7%), 57 CIN2 (10.6%), 67 (12.4%) CIN3 and 3 (0.6%) CIS. With regard to hrHPV status, women were classified as hrHPV positive (hrHPV+; *N* = 302; 56%) and hrHPV negative (hrHPV-; *N* = 237; 44%). The analysis of WBC LINE-1 methylation level was performed on women who provided blood sample for DNA analysis and the following results refer to this subgroup of women (*N* = 260). Notably, comparing women who provided blood samples with those who did not, no significant differences for socio-demographic and life-style factors were observed (data not shown).

Differences in WBC LINE-1 methylation levels, according to hrHPV status, were analyzed among women with normal cervical epithelium. Among the 252 women diagnosed with normal cervical epithelium, 96 had provided the blood sample for methylation analyses and were further classified as hrHPV- (*N* = 64) and hrHPV+ (*N* = 32). Table 1 displays the characteristics of women diagnosed with normal cervical epithelium according to hrHPV status. Particularly, the odds of being diagnosed with hrHPV infection increased among younger women (≤median age) (OR = 2.4; 95% CI = 1.0–5.8; *p* = 0.043), smokers (OR = 2.6; 95% CI = 1.1–6.2; *p* = 0.035), underweight-normal weight (OR = 3.2; 95% CI = 1.1–9.5; *p* = 0.028) and nulliparous women (OR = 4.9; 95% CI = 1.7–14.1; *p* = 0.002).

**Table 1:** Characteristics of healthy women according to hrHPV status

Characteristics	hrHPV+ (*n* = 32)	hrHPV- (*n* = 64)	*p-value* ^*a*^
Age (mean ± SD)	38.50 ± 9.54	42.39 ± 9.47	0.061
Smoking status (current)	50.0%	28.1%	**0.035**
BMI (mean ± SD)	22.26 ± 3.93	24.73 ± 5.31	**0.022**
Nutritional status			
Underweight	15.6%	3.1%	**0.030**
Normal weight	68.8%	59.4%	
Overweight	12.5%	20.3%	
Obese	3.1%	17.2%	
Parity (≥1 live births)	62.5%	89.1%	**0.002**
Education level (low)	37.5%	35.9%	0.881
Oral contraceptive use (yes)	12.5%	7.8%	0.458

Abbreviations: *SD* standard deviation, *BMI* Body Mass Index

^a^Statistically significant *p* values (*p* < 0.05) are indicated in bold font

Mean LINE-1 methylation level was 73.57% (median = 74.00%) and no significant difference was observed between hrHPV- and hrHPV+ women (Table 2). Results by multivariable logistic regression analysis showed that changes in LINE-1 methylation level were not associated with hrHPV status, adjusting for age, BMI, smoking status and parity (Table 3).

**Table 2:** Differences in LINE-1 methylation levels between hrHPV+ and hrHPV- women

LINE-1 methylation levels	hrHPV+ (*n* = 32)		hrHPV- (*n* = 64)		*p-value*
	Median	IQR	Median	IQR	
Site 1	67.00	17.00	71.00	18.00	0.498
Site 2	75.00	5.00	75.00	5.00	0.640
Site 3	77.00	6.00	76.00	3.00	0.913
Mean (all three sites)	73.50	3.83	74.33	4.25	0.407

Abbreviations: *LINE-1* Long Interspersed Nucleotide Element- 1, *IQR* Interquartile range

**Table 3:** Association between hrHPV status and LINE-1 methylation levels (logistic regression analysis adjusting for age, BMI, smoking status and parity)

	β (SE)	*p-value* ^a^	adjOR	95% CI	
				Lower	Upper
LINE-1 methylation level (continuous)	0.011 (0.047)	0.809	1.01	0.92	1.11
Age (continuous)	−0.014 (0.28)	0.633	0.97	0.93	1.04
BMI (continuous)	−0.076 (0.062)	0.220	0.93	0.82	1.05
Smoking status (current)	0.997 (0.493)	**0.043**	2.71	1.03	7.12
Parity (<1 live births)	1.302 (0.629)	**0.038**	3.68	1.07	12.61

Abbreviations: *SE* standard error, *adjOR* adjusted Odds Ratio, *CI* Confidence Interval, *LINE-1* Long Interspersed Nuclear Element- 1

^a^Statistically significant *p* values (*p* < 0.05) are indicated in bold font

Among the 302 hrHPV positive women, 139 have provided the blood sample for methylation analyses and were further classified as cases (*n* = 71; 51.1%), diagnosed as CIN 2 [*n* = 28], CIN 3 [*n* = 42] or CIS [*n* = 1], and controls (*n* = 68; 48.9%) including CIN 1 [*n* = 36] or normal cervical epithelium [*n* = 32].

Table 4 shows the characteristics of hrHPV+ women according to cases/controls classification. Taking into account socio-demographic variables and lifestyle factors, no statistically significant differences were observed between cases and controls. Mean LINE-1 methylation levels were 71.83 ± 10.20 (site 1), 74.28 ± 5.30 (site 2) and 76.91 ± 3.91 (site 3), respectively. No significant differences in LINE-1 methylation levels were observed according to age, BMI, smoking status, parity and oral contraceptive use (data not shown).

**Table 4:** Characteristics of hrHPV positive women according to cases/controls classification

Characteristics	Cases (*n* = 71)	Controls (*n* = 68)	*p-value*
Age (mean ± SD)	36.10 ± 7.88	37.84 ± 9.28	0.235
Smoking status (current)	49.3%	50.0%	0.934
BMI (mean ± SD)	22.89 ± 3.74	22.44 ± 3.63	0.470
Nutritional status			
Underweight	11.3%	11.8%	0.928
Normal weight	62.0%	66.2%	
Overweight	22.5%	19.1%	
Obese	4.2%	2.9%	
Parity (≥1 live births)	64.8%	54.4%	0.212
Education level (low)	46.5%	35.3%	0.180
Oral contraceptive use (yes)	14.1%	11.8%	0.684

Abbreviations: *SD* standard deviation, *BMI* Body Mass Index

**Fig. 1:**
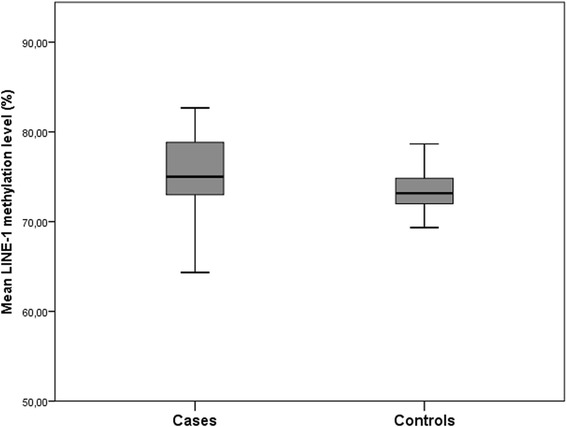
Methylation levels of LINE-1 in cases (CIN2+) and controls (≤CIN1). Mean methylation levels of LINE-1 sequences (mean percentage of methylation of the three evaluated CpG sites) in CIN2+ patients (cases) and in CIN 1 or normal cervical epithelium patients (controls) obtained using pyrosequencing of bisulfite converted DNA from WBCs (*p*-value = 0.002, based on the Mann-Whitney U test)

Table 5 and Fig. 1 show differences in LINE-1 methylation levels between cases and controls. Particularly, overall mean LINE-1 methylation level, and site 3, were higher in cases compared with controls (*p* = 0.002 and *p* = 0.032, respectively). Accordingly, logistic regression analysis showed a 1.1-fold increased odds of CIN2+ diagnosis associated with 1 unit increase in LINE-1 methylation level, adjusting for known factors related to LINE-1 methylation, such as age, BMI and smoking status (adjOR: 1.10; 95% CI:1.01–1.20; *p* = 0.032) (Table 6).

**Table 5:** Differences in LINE-1 methylation levels between cases and controls

LINE-1 methylation levels	Cases (*n* = 71)		Controls (*n* = 68)		*p-value* ^*a*^
	Median	IQR	Median	IQR	
Site 1	70.00	21.00	66.00	17.00	0.103
Site 2	76.00	4.00	75.00	5.00	0.090
Site 3	78.00	3.00	77.00	5.00	**0.032**
Mean (all three sites)	75.00	6.00	73.17	2.92	**0.002**

Abbreviations: *LINE-1* Long Interspersed Nuclear Element- 1, *IQR* Interquartile range

^a^Statistically significant *p* values (*p* < 0.05), based on the Mann-Whitney U test, are indicated in bold font

**Table 6:** Association between LINE-1 methylation level and case status (logistic regression analysis adjusting for age, BMI and smoking status)

	β (SE)	*p-value* ^a^	adjOR	95% CI	
				Lower	Upper
LINE-1 methylation level (continuous)	0.096 (0.045)	**0.032**	1.10	1.01	1.20
Age (continuous)	−0.030 (0.22)	0.178	0.97	0.93	1.01
BMI (continuous)	0.049 (0.051)	0.339	1.05	0.95	1.16
Smoking status (current)	−0.044 (0.351)	0.900	0.96	0.48	1.90

Abbreviations: *SE* standard error, *adjOR* adjusted Odds Ratio, *CI* Confidence Interval, *LINE-1* Long Interspersed Nuclear Element- 1

^a^ Statistically significant *p* values (*p* < 0.05) are indicated in bold font

**Fig. 2:**
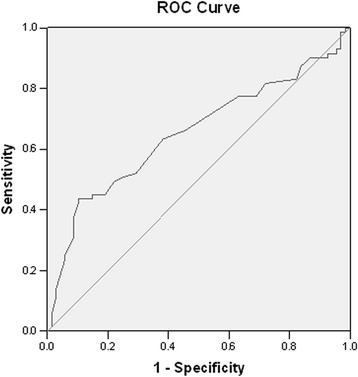
ROC curve analysis of LINE-1 methylation and CIN2+ detection. ROC (Receiver Operator Characteristics) curve of LINE-1 methylation levels for the detection of CIN2+. LINE-1 methylation level was suitable for detecting CIN2+ with an AUC of 0.652 (95% CI = 0.560–0.744). The cut-off value of 73.83% is the best threshold to separate cases from controls

To evaluate the performance of a model, based on LINE-1 methylation status, to distinguish cases from controls, an ROC curve analysis was performed. Figure 2 shows the ROC curve for detecting CIN2+ based on LINE-1 methylation level (AUC = 0.652, 95% CI = 0.560–0.744; *p* = 0.002). According to the definition of the minimum distance on the ROC curve from the (0,1) point (distance: 0.280), the cut-off value of 73.83% was the best threshold to separate cases from controls (sensitivity: 63.4% and specificity: 61.8%).

## Discussion

Identification of high-grade CIN lesions (CIN2+) by organized screening programs has shown high efficacy in reducing CC incidence and mortality worldwide [38, 39]. Since evidence from large randomized controlled trials demonstrated that hrHPV testing is more sensitive than cytology testing [40–44], the Italian Ministry of Health has recommended that regions shift toward HPV-based screening and has provided guidelines for its application [45, 46]. The identification of hrHPV+ women who are at risk of CIN2+ and CC and the validation of new biomarkers of disease progression are big challenges for the management of cervical abnormalities [46]. Particularly, the validation of blood-based methylation biomarkers is of great interest because they are easier to obtain and adaptable to population screening for the identification of cancer-affected individuals or those who are at higher risk of cancer. Among cancer patients and healthy controls, recent systematic reviews and meta-analyses have shown significantly different LINE-1 methylation levels in tissue samples [19], but not in blood leukocytes [19, 47]. We investigated whether LINE-1 methylation level in WBCs may represent a biomarker of cervical precursor lesions and cancer in hrHPV+ women. However, LINE-1 methylation has been investigated in several types of cancer and cannot be considered specific for CC. Furthermore, although the mechanisms leading to LINE-1 methylation changes in WBCs of cancer patients are currently uncertain, both LINE-1 hypomethylation and hypermethylation have been previously reported [21, 22, 32, 48–50].

Hypomethylation of repetitive elements which causes chromosomal instability is considered a molecular biomarker of cancer cells. Several studies have shown reduced LINE-1 methylation levels in cancer tissues and WBCs, especially in patients with head and neck, bladder and gastric cancer [27–32]. In contrast, other studies on bladder, renal, colorectal, ovarian, pancreatic cancers and cutaneous melanoma have reported higher LINE-1 methylation levels in WBCs of cancer patients [20–26]. A plausible explanation for this relationship is that LINE-1 sequences with double strand DNA breaks had higher methylation levels around the area of the break, compared to DNA without double strand breaks [51]. Thus, the DNA damage and the increased frequency of double strand DNA breaks in non-healthy individuals could explain the hypermethylation in WBCs DNA.

At the best of our knowledge, only the study by Piyathilake et al. [8] has currently evaluated the association between LINE-1 methylation and CIN2+ status, in blood samples. The degree of LINE-1 methylation was lower in high grade CIN patients (mean = 63% ± 7%) than in controls (mean = 64% ± 7%), albeit difference was small. Particularly, the risk to be diagnosed with CIN2+ was lower among women in the highest tertile of LINE-1 methylation level (≥70%), compared to women in the lower tertiles [8]. To support this association, the authors assumed that higher LINE-1 methylation levels could mediate a positive effect on immune response against HPV infection [8]. However, an in vitro study on squamous cell carcinoma cell lines revealed higher LINE-1 methylation level in HPV+ compared to HPV- cells [52]. This result partially confirmed the positive correlation between the maintenance of normal LINE methylation and HPV-positivity, observed by Richards et al. in head and neck cancer tissues and cell lines [33].

Accordingly, in order to investigate the potential association between WBC LINE-1 methylation level and hrHPV status, we analyzed women with normal cervical epithelium, to avoid the possibility of reverse causation mediated by the carcinogenic process (i.e. the degree of LINE-1 methylation could be influenced by the carcinogenic process). Results of our study showed that LINE-1 methylation levels were not different between hrHPV+ and hrHPV- women. Besides, the degree of LINE-1 methylation was not associated with hrHPV status, also taking into account hrHPV related variables such as age, BMI, smoking status and parity. However, additional studies are required to assess the role of LINE-1 methylation in cell-mediated response to HPV infection.

Among hrHPV+ women, we were able to show that WBC LINE-1 methylation level was higher in subjects diagnosed with CIN2+ (median = 75.00%; IQR = 73.00%–79.00%), compared to healthy women and those with low grade cervical lesions (median = 73.17%; IQR = 72.00%–75.33%).

This small, but statistically significant, difference in LINE-1 methylation levels could be due to factors that influence the association between DNA methylation and cancer risk [53]. For example, previous studies have shown that global hypomethylation can occur with increasing age [54, 55].

Since, in the present study, cases were younger than controls, we analysed whether LINE-1 methylation levels were different according to age. Consistently with results from previous studies [28, 56–59], we did not observe association between age and LINE-1 methylation levels in WBCs DNA. Moreover, on the basis of a multivariable model, the association between LINE-1 methylation and CIN2+ did not depend on age, BMI, and smoking status. Particularly, for a one-unit increase in LINE-1 methylation level, the odds of being diagnosed with CIN2+ increased by 10% (adjOR = 1.10; 95% CI:1.01–1.20), adjusting for age, BMI, and smoking status. Thus, the odds of being diagnosed with CIN2+ looked to be slightly associated with LINE-1 methylation status. However, the retrospective nature of our study did not make it possible to establish whether the increase in LINE-1 methylation level was a cause or a consequence of tumor progression. Moreover, although the present study did not show evidence of association between LINE-1 methylation and other socio-demographic and life-style factors, the contribution of other unmeasured variables cannot be excluded. Particularly, previous studies have reported the influence on LINE-1 methylation levels of *MTHFR* polymorphisms [60], diet, nutrient intakes, folate deficiency [35] and amount of physical activity [61]. Thus, future studies should consider other influential factors to confirm the present findings.

In order to evaluate the potential use of LINE-1 methylation as a biomarker for CC risk, the optimal cut-off value, suitable to distinguish cases from controls, has been assessed through an ROC curve analysis. Our results demonstrate that a model based on LINE-1 methylation level had limited performance for the diagnosis of CIN2+ lesions, with moderate sensitivity (63.4%) and specificity (61.8%). Moreover, the cut-off value (73.8%), obtained from the ROC curve analysis, is very close to median value of LINE-1 methylation in hrHPV+ healthy controls (73.4%). Thus, results from ROC curve analysis do not encourage the use of LINE-1 methylation as a stand-alone blood-based biomarker for CC risk. Its potential clinical value for the screening of women at risk of CC needs to be evaluated by large prospective studies and randomized controlled trials, which take into account tumor progression through pre-neoplastic lesions.

However, a potential goal for the future would be that a novel methylation biomarker panel, using LINE-1 methylation status and other differentially methylated regions [62–64], could be proposed and validated for the screening of women at risk of CC.

Strengths of this study consist in the use of protocols and methodologies for blood collection, DNA extraction and DNA methylation analysis consistent between cases and controls. Moreover, to investigate difference and variability in LINE-1 methylation levels within histological groups, data were analysed with a robust statistical approach. The potential effect of hrHPV infection on WBC LINE-1 methylation level was investigated in women with normal cervical epithelium, also taking into account hrHPV related risk factors, through a multivariable logistic regression model. The degree of LINE-1 methylation was not associated with hrHPV status, even though we were not able to stratify the effect for specific hrHPV types (i.e. HPV16, HPV18 and others).

As reported by the previous contrasting study [8], difference in LINE-1 methylation levels between cases and controls was modestly different. A multivariable logistic regression model was applied to adjust our result for factors that are commonly known to affect methylation biomarkers. Conversely to previously published results [8], independent variables (i.e. LINE-1 methylation level, age and BMI) were entered in the regression model as continuous variables, to avoid considerable loss of statistical power and residual confounding caused by dichotomization of continuous variables [65]. This makes more accurate the interpretation of the coefficient of LINE-1 methylation level in the regression model, being able to partially explain controversial findings.

With regard to molecular analysis, precision and reproducibility of the DNA methylation assay are very important characteristics to assess the utility of LINE-1 methylation as a biomarker in clinical practice. High reliability and flexibility have made pyrosequencing of bisulfite-treated DNA the “gold standard” [66, 67], and a high-throughput and replicable methodology to evaluate LINE-1 methylation as a surrogate marker for global DNA methylation [66–70]. Furthermore, several studies have reported that pyrosequencing has good precision at higher methylation levels, and can provide a reliable measure of LINE-1 methylation in WBC DNA [71–76]. Particularly, results by Iwagami et al. [77] indicate that run-to-run variation of LINE-1 methylation degrees is not large, and a single run of PCR pyrosequencing can provide reasonably precise measures.

Additional important issues should be considered when interpreting results of the present study. Firstly, LINE-1 methylation levels can vary depending on the target CpG site and on the tissue type [68, 69]. The distinctiveness of LINE-1 methylation levels discourages the comparison between results from studies which evaluate LINE-1 methylation status at different CpG sites [29]. Since CpG sites analysed in the present study differ from those analysed in others, this could partially explain both the discrepancies with findings reported by Piyathilake et al. [8] and also the high variability in LINE-1 methylation levels among our population, when compared to previously published studies [20, 22].

Recent results report the variability of methylation degree of LINE-1 sequences. It has been reported that repetitive elements, including LINE-1 and Alu, are strongly hypomethylated in epithelial ovarian cancer tissue as compared to the normal tissue of control subjects. Conversely, WBCs DNA of cancer patients was hypermethylated compared to controls, suggesting that the mechanisms controlling global methylation in cancer and in normal tissues are distinct [24]. Secondly, previous studies have reported that differences in blood cell composition could lead to variation in methylation levels [70]. In our study, DNA was extracted from whole blood and differences in the proportion of blood cell subtypes could represent a limitation of this study, reinforcing the importance of accounting for cellular heterogeneity in clinical practice and research [26].

Finally, to detect methylation changes and variability, an exhaustive investigation of the relationship between LINE-1 DNA methylation and CC risk would require the study of a large cohort of prospectively collected blood samples.

## Conclusions

Although several previous studies have investigated the association between WBCs DNA methylation levels and cancer, to the best of our knowledge, our study is the first to identify an association between LINE-1 hypermethylation and CIN2+. LINE-1 methylation status in WBCs may represent a cost-effective and tissue-accessible biomarker for high-grade CIN in hrHPV positive women. However, a model based solely on LINE-1 methylation levels has limited performance and other investigations are necessary to further elicit the role of WBCs DNA methylation in CC. As a result, LINE-1 methylation in WBCs could be proposed as a target in a novel methylation biomarker panel, based on differentially methylated regions, for non-invasive early diagnosis in women at risk of CC. However, genome-wide analyses to identify differentially methylated regions and further validation of potential markers through a systematic approach should be encouraged.

## Abbreviations

95% CIs: 95% confidence intervals
AUC: Area Under the Curve
BMI: Body mass index
CC: Cervical cancer
CIN: Cervical Intraepithelial Neoplasia
CIS: carcinoma in situ
hrHPV: high risk Human Papilloma Virus
IQRs: interquartile ranges
LINEs-1: Long Interspersed Nuclear Elements - 1
ORs: odds ratios
ROC: Receiver-Operating Characteristic
SDs: standard deviations
WBCs: white blood cells
